# Data on xylem sap proteins from Mn- and Fe-deficient tomato plants obtained using shotgun proteomics

**DOI:** 10.1016/j.dib.2018.01.034

**Published:** 2018-02-03

**Authors:** Laura Ceballos-Laita, Elain Gutierrez-Carbonell, Daisuke Takahashi, Anunciación Abadía, Matsuo Uemura, Javier Abadía, Ana Flor López-Millán

**Affiliations:** a Plant Stress Physiology Group, Plant Nutrition Department, Aula Dei Experimental Station, CSIC, P.O. Box 13034, 50080 Zaragoza, Spain; b United Graduate School of Agricultural Sciences, Iwate University, Morioka 020-8550, Japan; c Cryobiofrontier Research Center, Faculty of Agriculture, Iwate University, Morioka 020-8550, Japan; d USDA-ARS Children's Nutrition Research Center, Department of Pediatrics, Baylor College of Medicine, 1100 Bates St., Houston, TX 77030, USA

**Keywords:** Iron deficiency, Mn deficiency, Proteomics, Shotgun proteomics, Tomato, Xylem sap

## Abstract

This article contains consolidated proteomic data obtained from xylem sap collected from tomato plants grown in Fe- and Mn-sufficient control, as well as Fe-deficient and Mn-deficient conditions. Data presented here cover proteins identified and quantified by shotgun proteomics and Progenesis LC-MS analyses: proteins identified with at least two peptides and showing changes statistically significant (ANOVA; *p* ≤ 0.05) and above a biologically relevant selected threshold (fold ≥ 2) between treatments are listed. The comparison between Fe-deficient, Mn-deficient and control xylem sap samples using a multivariate statistical data analysis (Principal Component Analysis, PCA) is also included. Data included in this article are discussed in depth in the research article entitled “Effects of Fe and Mn deficiencies on the protein profiles of tomato (*Solanum lycopersicum)* xylem sap as revealed by shotgun analyses” [1]. This dataset is made available to support the cited study as well to extend analyses at a later stage.

**Specifications Table**

**Table:** 

Subject area	Biology
More specific subject area	Plant Physiology
Type of data	Tables, figures and images (pictures of plant material)
How data was acquired	Shotgun mass spectroscopy approach using an ADVANCE UHPLC system
Data format	Raw, statistical uni- and multi-variate analysis
Experimental factors	Proteins were directly isolated from the xylem sap of Fe-deficient, Mn-deficient and control (Fe- and Mn-sufficient) plants
Experimental features	Plants grown in nutrient solution under control, Fe- and Mn-deficient conditions were used and xylem sap collected by de-topping. Xylem sap proteins were precipitated, resuspended and analyzed by label free LC-MS/MS [1].
Data source location	Tomato (*Solanum lycopersicum*, cv. Tres Cantos) plants were grown hydroponically in a controlled environment chamber
Data accessibility	The MS proteomics data have been deposited to the ProteomeXchange Consortium *via* the Pride partner repository with the data set identifier PXD007517.

**Value of the data**

• Tomato xylem sap proteins identified and quantified using a shotgun approach are presented herein, providing data on the protein composition of this fluid and facilitating comparisons with other plant species and different plant stresses.
• Statistically significant and biologically relevant changes in the xylem sap protein composition upon Fe and Mn deficiencies provide information to assess the effects of these nutritional deficiencies on the metabolic pathways of tomato plants growing in controlled environmental conditions, and results could be compared to those found in other nutritional constraints.
• Data would allow other researchers to assess the companion paper [1], extend analyses at a later stage and facilitate the study of target proteins in tomato xylem sap.

## 1 Data

**Fig. 1:**
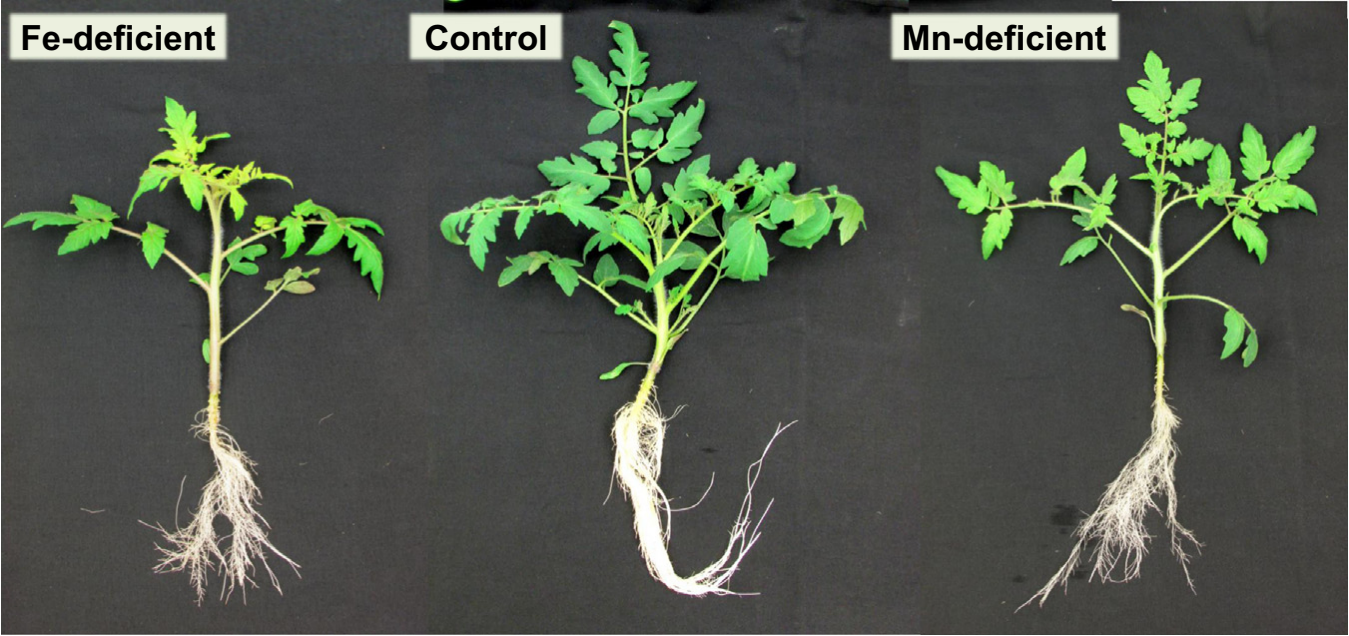
Composite image depicting pictures of whole plants from Fe-deficient, control and Mn-deficient treatments at the time of sampling.

**Fig. 2:**
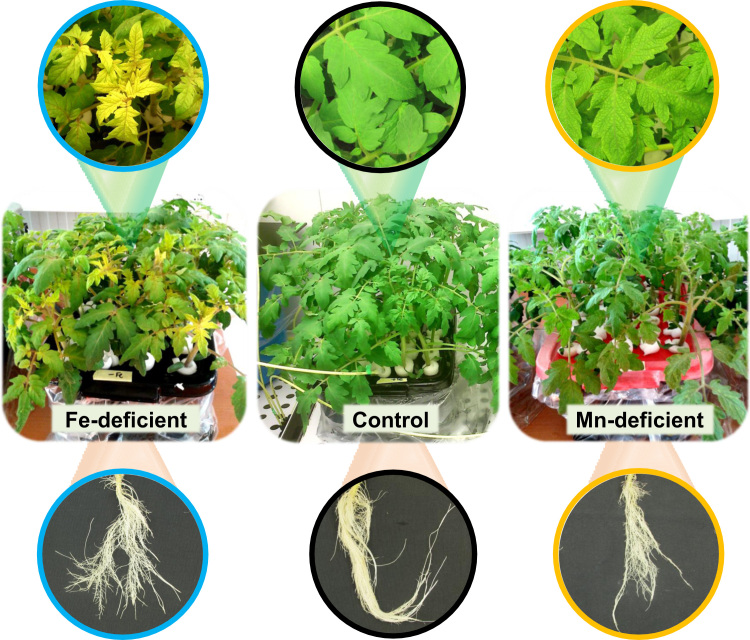
Composite image showing pictures of the plant culture set up for Fe-deficient, control and Mn-deficient plants at the time of sampling, with insets showing close-ups of the leaves and roots of the same plants.

**Fig. 3:**
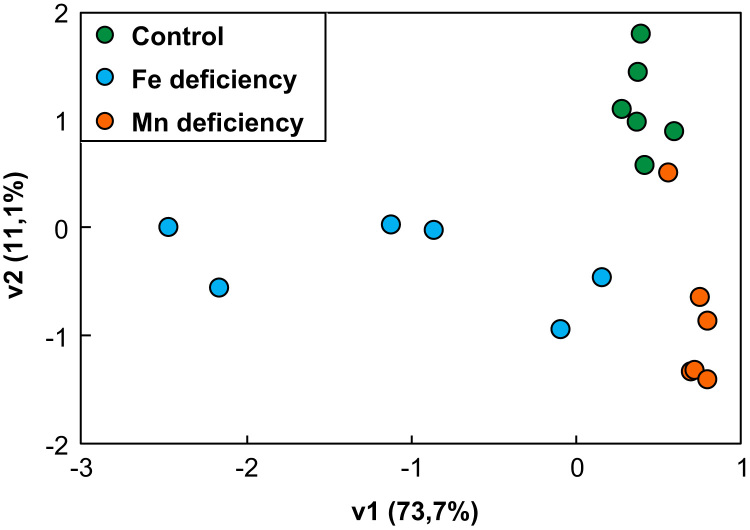
Score scatter PCA (Principal Component Analysis) plot of nutrient-deficient samples when compared to controls. Principal component analysis was carried out using SPSS Statistical software (v. 24.0) and included proteins showing statistically significant changes (ANOVA; *p* < 0.05) as a result of the nutrient deficiency treatments. Control, Fe-deficient and Mn-deficient samples are depicted with green, blue and orange circles, respectively.

The proteome data presented herein were collected from xylem sap fluid of tomato plants grown in two nutritional deficiencies that occur often in plants, Fe and Mn deficiency (Fig. 1, Fig. 2). A shotgun proteomic approach and data processing software were used to identify and quantify a large number of proteins in the xylem sap as well as to assess the changes induced in the proteome of this fluid by these nutritional stresses. The peptides used in the quantification and the protein profiling of the xylem sap proteome are shown in Tables S1 and S2, respectively. To assess the effects of Fe-deficiency or Mn-deficiency, the ratios of normalized protein abundances between nutrient-deficient and control samples were calculated, and proteins showing changes statistically significant (ANOVA; *p* ≤ 0.05) and above a biologically relevant threshold (fold ≥ 2) are shown in Tables S3 and S4, respectively. Multivariate statistical analyses (Principal Component Analysis, PCA) of proteins showing statistically significant changes (ANOVA; *p* < 0.05) are shown in Fig. 3. The MS proteomics data have been deposited to the ProteomeXchange Consortium *via* the Pride partner repository with the data set identifier PXD007517. The full description of Materials and Methods, Results and Discussion for this data set are presented in the associated research article [1].

## 2 Experimental design, materials and methods

For each of three treatments (Fe- and Mn-sufficient control, Fe-deficient and Mn-deficient conditions), the xylem sap fluid from six independent batches of plants were collected after eight days of treatment (Fig. 1, Fig. 2). In each batch of plants, fluid from 16–18 plants was pooled together and considered as a biological replicate (total *n* = 6). Xylem sap proteins were precipitated, resuspended in the appropriate buffer [1] and analyzed by shotgun proteomics. Mass spectrometry analysis was carried out on an LTQ Orbitrap XL (Thermo Fisher Scientific, Waltham, MA, U.S.A.) equipped with Xcalibur software (v. 2.0.7, Thermo Fisher Scientific). Parameters used were: peptide mass and MS/MS tolerances of ± 5 ppm and 0.6 Da, respectively; one missed cleavage, fixed modification carbamidomethylation (Cys) and variable modification oxidation (Met) allowed; and peptide charges + 1 to + 3. Data files obtained were processed (Progenesis QI) and all peptides identified and quantified are shown in Table S1. Proteins identified (MASCOT v. 2.4.1 using the NCBI database) are shown in Table S2. Data on abundance changes between treatments are provided after using two filters: ANOVA statistical significance (*p* ≤ 0.05) and a biologically relevant threshold level (fold ≥ 2) (Tables S3 and S4, for Fe and Mn deficiencies, respectively). Principal Component Analysis (PCA) analyses were carried out using SPSS Statistical software (v. 24.0) (Fig. 3).

Protein identification was carried out using the list of total peptides with the Mascot search engine [1]. Protein information was exported in Mascot.xml format and imported to Progenesis [1], which then associated peptide and protein information.

Positive protein identification was assigned with at least two unique top-ranking peptides matched and with scores above the statistical and biological threshold levels (ANOVA; *p* ≤ 0.05 and fold ≥ 2). Proteins achieving these thresholds are shown in Tables S3 and S4, for Fe-and Mn-deficient samples, respectively.

## Appendix A Supplementary material

Supplementary material Table S1: List of peptides identified and quantified by shotgun proteomics and Progenesis LC-MS analyses.

.

Supplementary material Table S2: List of proteins identified and quantified by shotgun proteomics and Progenesis LC-MS analyses. Table S3: List of proteins showing significant changes (ANOVA; *p* ≤ 0.05) above the biological relevance threshold (fold ≥ 2) and identified and quantified with at least two peptides when Fe-deficient xylem sap samples were compared to those of control conditions. Table S4: List of proteins showing significant changes (ANOVA; *p* ≤ 0.05) above the biological relevance threshold (fold ≥ 2) and identified and quantified with at least two peptides when Mn-deficient xylem sap samples were compared to those of control conditions.

## References

[1] L. Ceballos-Laita, E. Gutierrez-Carbonell, D. Takahashi, A. Abadía, M. Uemura, J. Abadía, A.F. López-Millán. Effects of Fe and Mn deficiencies on the protein profiles of tomato (Solanum lycopersicum) xylem sap as revealed by shotgun analyses. *J. Proteomics* (2018) 170, 117-129, 10.1016/j.jprot.2017.08.018 10.1016/j.jprot.2017.08.018 28847647

